# Annotated bacterial chromosomes from frame-shift-corrected long-read metagenomic data

**DOI:** 10.1186/s40168-019-0665-y

**Published:** 2019-04-16

**Authors:** Krithika Arumugam, Caner Bağcı, Irina Bessarab, Sina Beier, Benjamin Buchfink, Anna Górska, Guanglei Qiu, Daniel H. Huson, Rohan B. H. Williams

**Affiliations:** 1 Singapore Centre for Environmental Life Sciences Engineering, Nanyang Technological University, 60 Nanyang Drive, SBS-01N-27, Singapore, 637551 Singapore; 2 Department of Computer Science, University of Tübingen, Sand 14, Tübingen, 72076 Germany; 3 International Max Planck Research School From Molecules to Organisms, Max Planck Institute for Developmental Biology and Eberhard Karls University Tübingen, Max-Planck-Ring 5, Tübingen, 72076 Germany; 4 Singapore Centre for Environmental Life Sciences Engineering, National University of Singapore, 28 Medical Drive, Singapore, 117456 Singapore; 5 Max-Planck-Institute for Developmental Biology, Max-Planck-Ring 5, Tübingen, 72076 Germany; 6 Life Sciences Institute, National University of Singapore, 28 Medical Drive, Singapore, 117456 Singapore

**Keywords:** Microbiome, Long-read sequencing, Microbial genomics, Sequence assembly, Frame-shifts, Algorithms, Software

## Abstract

**Background:**

Short-read sequencing technologies have long been the work-horse of microbiome analysis. Continuing technological advances are making the application of long-read sequencing to metagenomic samples increasingly feasible.

**Results:**

We demonstrate that whole bacterial chromosomes can be obtained from an enriched community, by application of MinION sequencing to a sample from an EBPR bioreactor, producing 6 Gb of sequence that assembles into multiple closed bacterial chromosomes. We provide a simple pipeline for processing such data, which includes a new approach to correcting erroneous frame-shifts.

**Conclusions:**

Advances in long-read sequencing technology and corresponding algorithms will allow the routine extraction of whole chromosomes from environmental samples, providing a more detailed picture of individual members of a microbiome.

**Electronic supplementary material:**

The online version of this article (10.1186/s40168-019-0665-y) contains supplementary material, which is available to authorized users.

## Background

Second-generation sequencing has been the work-horse of metagenomic analysis of microbiomes, with typical studies based on hundreds of millions of short reads [1, 2]. While the taxonomic and functional binning of short metagenomics read data are reasonably straight-forward computational problems [3], much recent work has focused on the challenge of assembling and binning metagenomic contigs, a procedure which provides invaluable working models of the genomes of member species [4]. However, the assembly of whole bacterial chromosomes from short metagenomic reads has proven to be an all but impossible task.

Third generation sequencing promises to allow the extraction of whole genomes from environmental samples with ease [5]. This promise is now beginning to be fulfilled. Here, we report on the results of a single ONT MinION run on a microbial community from an enrichment bioreactor targeting polyphosphate accumulating organisms (PAO), that had been inoculated with activated sludge from a full-scale water reclamation plant in Singapore.

## Results

**Fig. 1:**
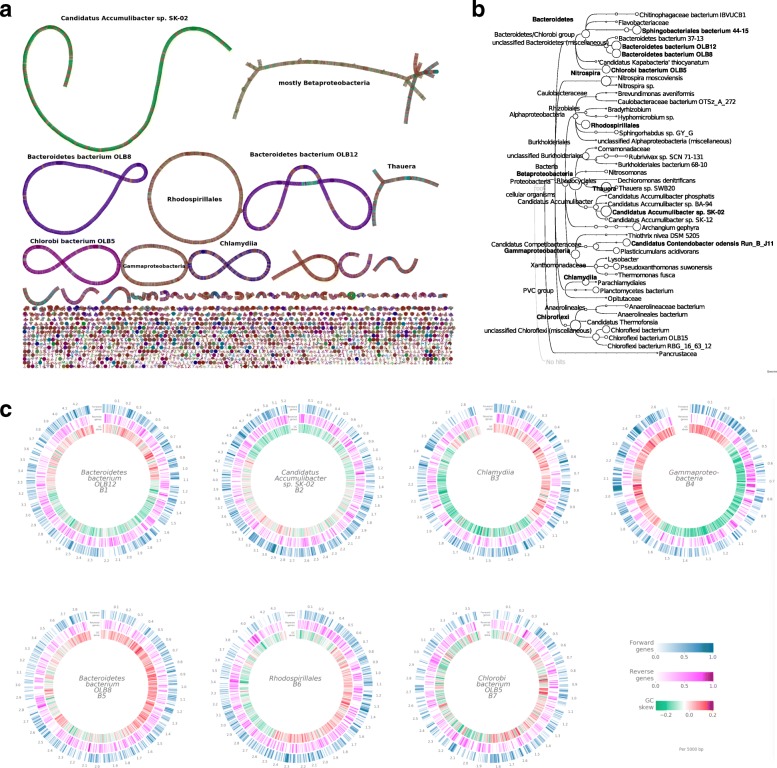
Summary of results. **a** Bandage [30] visualization of the Unicycler assembly graph before final segmentation into contigs. The largest connected components are labeled by the corresponding taxonomic bins, and the nodes are colored by the MEGAN taxonomic classification of the corresponding long reads. The seven longest linear and circular components correspond to the seven LR-chromosomes. **b** MEGAN-LR taxonomic binning: nodes are scaled to indicate the number of aligned bases in each bin. Bins that are more than 50% complete are shown in bold. **c** Annotation of the seven LR-chromosomes, labeled by the corresponding taxonomic bins. The three circular tracks indicate the genes annotated by Prokka on the forward strand (blue) and reverse strand (pink), and GC-skew (green and red indicate lower or higher than average GC content, respectively)

Running a MinION sequencer for 1 day, we obtained ≈695,000 long reads with an average length of 9 kb, totaling approximately 6 Gb of sequence (Additional file 1: Table S1). Using Unicycler [6–8], we assembled these into 1702 contigs (LR contigs) of average length 61 kb (Additional file 2: Table S2). We observed 10 contigs over 1 Mb in length, including five circular contigs between 2.7 and 4.2 Mb long (see Fig. 1a). In principle, long-read assembly procedures could generate complete genomes *de novo*, without the need for complex contig binning procedures, and accordingly we designed tools and analyses to determine the extent to which such long contigs represented genomes of member species of the community. Our analyses are based on (1) the analysis of genome completeness and quality, (2) whole genome comparisons to reference genomes, and (3) comparison with metagenome-assembled genomes recovered from short reads sequenced from the same DNA sample.

Long reads, and, to a lesser degree, LR contigs, suffer from a high rate of erroneous insertions and deletions, which lead to frame-shifts in translated alignments. For the data presented here, the average number of frame-shifts per kilobyte of aligned sequence is 14.8, for unassembled long reads, and 6, for LR contigs, with a standard deviation of 2.9 and 2, respectively. For this reason, genome evaluation tools (such as CheckM [9]) and annotation workflows (such as Prokka [10]), which typically employ translated alignments, perform poorly on current long-read data.

**Fig. 2:**
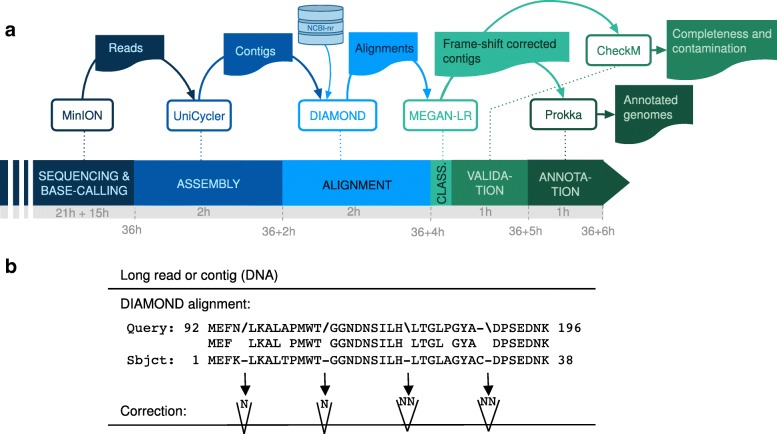
Analysis. **a** Long-read analysis pipeline shown from left to right. MinION sequencing produces a set of reads. These are assembled into contigs using Unicycler and aligned against the NCBI-nr database using DIAMOND. The contigs and alignments are processed by MEGAN so as to perform taxonomic binning and also to produce frame-shift-corrected contigs. These are analyzed using CheckM and annotated using Prokka. The duration of each step is shown in wall-clock hours. MEGAN analysis took less than 10 min. **b** Frame-shift correction: in frame-shift alignments, forward slashes, and backward slashes indicate a frame decrease, or increase, by one, respectively. Correction is performed by inserting one or two unspecified nucleotides into the sequence, respectively

To address this deficiency, we have developed a two-step frame-shift correction technique. First, we have modified DIAMOND [11] (v 0.9.23) so as to perform a *frame-shift aware* DNA-to-protein alignment [12] of the sequences against the NCBI-nr protein reference database [13]. Second, based on the location of frame-shifts reported in the alignments, we insert Ns into the sequences so as to maintain the frame (see Fig. 2b). Sequences corrected in this way can be evaluated and annotated using conventional genome quality and annotation tools.

We performed initial taxonomic analysis of all LR contigs using MEGAN-LR [14] (v 6.13.3), obtaining 106 taxonomic bins at different taxonomic ranks (see Fig. 1b and Additional file 3: Table S3). To determine whether these taxonomic bins might harbor complete genomes, we applied CheckM to the set of frame-shift-corrected LR contigs contained in each taxonomic bin. This analysis indicates that 14 of the bins are more than 50% complete. Of these, six fulfill the definition of a “high quality draft” metagenome-assembled genome (namely, completeness >  90*%* and contamination <  5*%*). For purposes of this paper, we also consider the seventh bin listed in Table 1 as high quality, as it consists of only one circular LR contig and is of chromosomal length. There are four additional bins that reach the level of “medium quality draft” (completeness >  50*%* and contamination <  10*%*) [15].

**Table 1:** Summary of results

	(a)	(b)	(c)	(d)	(e)	(f)	(g)	(h)	(i)	(j)
	DIAMOND+MEGAN	Unicycler	Total	Aligned	Average	CheckM	Prokka			
	taxonomic bin	contigs	(Mb)	(Mb)	coverage	Complete.	contam.	rRNA	tRNA	CDS
High-quality draft genomes:										
B1	*Bacteroidetes bacterium* OLB12	1	4.2	3.5	57.3	95%	0.1%	6	39	4,163
B2	*Candidatus Accumulibacter* SK-02	1	5.2	4.1	384.2	94%	0.6%	4	53	4,915
B3	*Chlamydiia* (class)	1	2.8	1.8	48.8	94%	2%	6	39	3,387
B4	*Gammaproteobacteria* (class)	43	4.7	3.0		93%	2%	6	52	4,833
	-Longest contig		2.7	1.6	25.1	93%	0.2%	3	40	3,359
B5	*Bacteroidetes bacterium* OLB8	1	3.8	3.0	52.1	93%	1%	6	37	3,394
B6	*Rhodospirillales* (order)	1	4.4	3.0	29.5	92%	0.5%	3	47	4,015
B7	*Chlorobi bacterium* OLB5	1	3.5	2.5	38.7	88%	1%	3	41	4,131
Medium quality draft genomes:										
B8	*Thauera* (genus)	25	4.6	4.0		89%	4%	12	64	4,040
	-Longest contig		0.8	0.7	32.7	14%	0%	0	5	672
B9	*Sphingobacteriales bacterium* 44-15	59	3.2	2.8		76%	1%	2	17	2,953
	-Longest contig		0.2	0.1	10.2	0%	0%	0	0	172
B10	*Bacteroidetes* (phylum)	43	3.9	2.6		72%	7%	1	12	1,997
	-Longest contig		1.2	0.8	14.1	32%	0%	0	3	807
B11	*Candidatus Contendobacter* B J11	39	2.5	2.0		59%	9%	2	37	2,668
	-Longest contig		0.3	0.3	15.4	19%	0%	0	7	295
Low quality draft genomes:										
B12	*Betaproteobacteria* (class)	111	6.6	5.5		89%	79%	6	71	4,655
	-Longest contig		0.4	0.3	37.1	10%	0%	0	1	372
B13	*Nitrospira* (genus)	34	4.2	3.7		83%	13%	0	6	563
	-Longest contig		1.1	0.9	17.6	27%	0%	0	2	99
B14	*Chloroflexi* (phylum)	151	5.4	4.3		71%	29%	0	11	3,565
	-Longest contig		0.2	0.2	13.3	8%	0%	0	1	86

For all 14 taxonomic bins B1–B14 that CheckM deems ≥ 50*%* complete (a), and -in cases where the bin contains more than one contig- also for the longest contig, in descending order of assembly quality, we report (b) the number of contigs produced by Unicycler, (c) the total number of bases, (d) the number of bases aligned by DIAMOND to some protein reference, (e) the average coverage by long reads (based on the longest contig), (f) the %-completeness and (g) %-contamination reported by CheckM, and (h)–(j), the number of rRNA, tRNA and coding sequences reported by Prokka, respectively

In all seven high-quality bins, the CheckM results derive from a single long contig, of length 2.7−5.2 Mb, with the numbers of cognate rRNA and tRNA genes, and protein coding genes, as reported by Prokka, all lying within the range usually seen for bacterial genomes (see Table 1 and Additional file 3: Table S3). Throughout this paper, we will refer to these long contigs as the seven *LR chromosomes*.

**Fig. 3:**
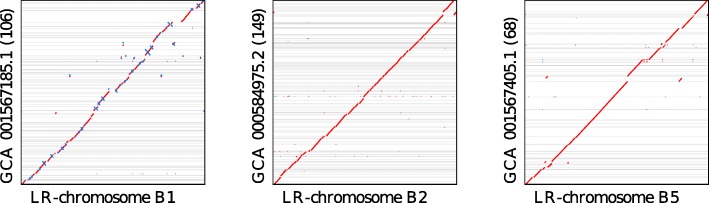
Dot plots for the three LR-chromosomes that have high similarity to reference genome assemblies, namely, B1 against GCA_001567185.1 (*Bacteroidetes bacterium* OLB12), B2 against GCA_000584975.2 (*Candidatus accumulibacter* sp. SK-02), and B5 against GCA_001567405.1 (*Bacteroidetes bacterium* OLB8). Forward alignments are shown in red, whereas reverse complemented alignments are shown in blue, and gray lines indicate contig boundaries in the reference assemblies. The number of contigs in each reference sequence is given in brackets

From the seven high quality taxonomic bins, we obtained a near-complete LR chromosome (number B2 in Table 1) that is binned to *Candidatus Accumulibacter*, a polyphosphate accumulating organism (PAO) that is commonly observed in waste-water treatment plants and is the target of our enrichment protocol [16]. Two circular LR chromosomes (B1 and B5) are binned to the species *Bacteroidetes bacterium* OLB8 and OLB12, both of which were originally recovered as metagenome-assembled genomes from a partial-nitritation anammox (PNA) bioreactor community, where they are thought to function as aerobic heterotrophs [17]. All three of these LR-chromosomes align end-to-end to their corresponding (fragmented) reference genomes (see Fig. 3).

The remaining four are closed circular chromosomes that do not align to any current reference genome and thus most likely represent novel organisms. One of these (B3) is binned to the class of *Chlamydiia*. Although normally considered an obligate intracellular pathogen in humans, members of the phylum *Chlamydiae* are known to occur in microeukaryotes that occur as predators in such reactor communities [18]. Another (B6) is binned to *Rhodospirillales* and contains a 16S sequence that maps to the genus *Defluviicoccus*. Some members of this genus compete with PAO for carbon sources and are commonly observed in PAO enrichment reactors [19]. Another LR chromosome (B4) is binned to the class *Gammaproteobacteria*. Finally, we obtained an LR chromosome (B7) that is binned to *Chlorobi bacterium OLB5*, an organism previously observed in waste-water [17].

For all seven LR-chromosomes, Silva analysis [20] of the contained 16S sequences confirm the taxon bin assignment obtained by MEGAN analysis (see Table 2).

**Table 2:** For all seven LR chromosomes, we list the MEGAN and Silva taxonomic assignments

Bin	MEGAN assignment	Silva assignment
B1	*Bacteroidetes bacterium* OLB12	*Bacteroidetes; Bacteroidia; Cytophagales; Microscillaceae;* OLB12
B2	*Candidatus Accumulibacter* sp. SK-02	*Proteobacteria; Gammaproteobacteria; Betaproteobacteriales; Rhodocyclaceae; Candidatus Accumulibacter*
B3	*Chlamydiia* (class)	*Chlamydiae; Chlamydiae; Chlamydiales; Parachlamydiaceae*
B4	*Gammaproteobacteria* (class)	*Proteobacteria; Gammaproteobacteria; Coxiellales; Coxiellaceae; Coxiella*
B5	*Bacteroidetes bacterium* OLB8	*Bacteroidetes; Bacteroidia; Chitinophagales; Saprospiraceae;* OLB8
B6	*Rhodospirillales* (order)	*Proteobacteria; Alphaproteobacteria; Rhodospirillales; Rhodopirillaceae; Defluviicoccus*
B7	*Chlorobi bacterium* OLB5	*Ignavibacteriae; Ignavibacteria; Ignavibacteriales; Ignavibacteriaceae*

Solely for the purpose of verification, we also produced a second independent set of paired reads from the same DNA aliquot using Illumina short-read sequencing. First, we used the short-read clone coverage to detect potential break-points in the assemblies of 7 LR chromosomes that might indicate long-read assembly errors, and found 11. All but one of these positions have very good long-read coverage, making an assembly error unlikely at these positions. Second, we assembled the short reads and aligned the short-read contigs against the long-read contigs, and this comparison shows a very high degree of co-linearity within the SR contigs (see Fig. 5). Third, we performed metagenomic binning of the short-read contigs and compared the short-read bins with the long-read chromosomes, confirming a very high level of concordance between the two assemblies (see Fig. 6 and Additional file 11: Figure S3).

**Fig. 4:**
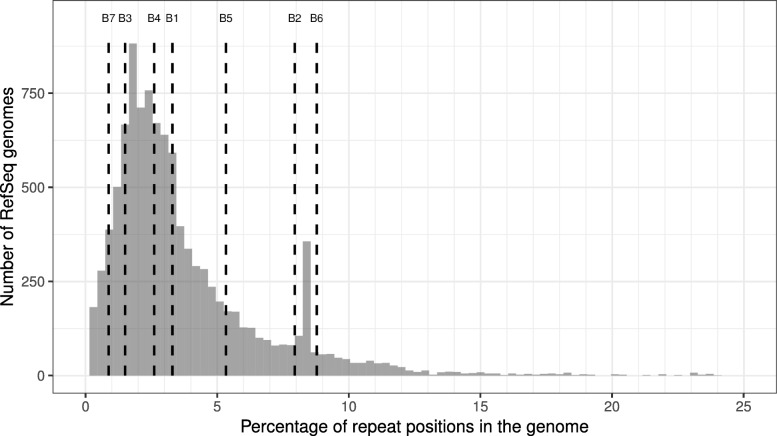
Distribution of repeat rates in all complete bacterial genomes in RefSeq. Vertical lines show the repeat rate of the seven LR chromosomes. Additional 17 data points that have a repeat percentage above 25% are not shown

**Fig. 5:**
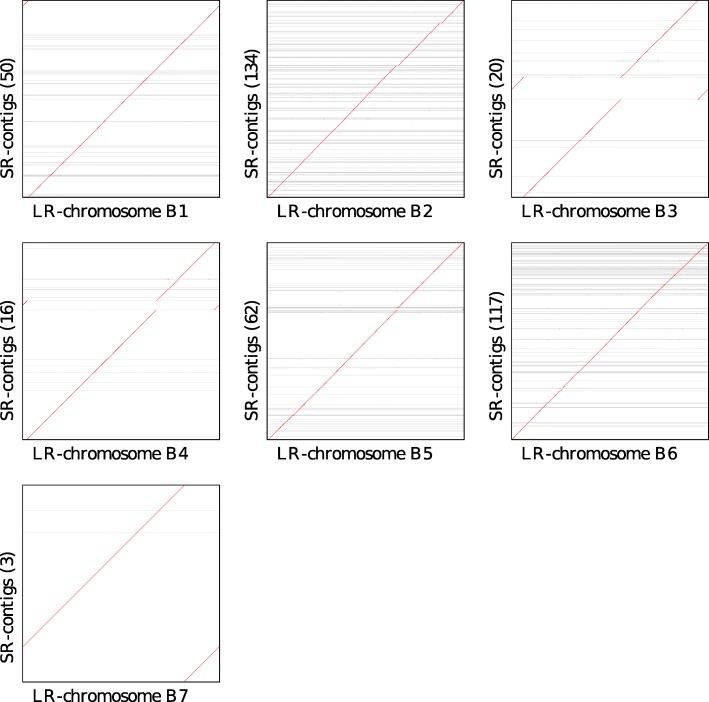
For each of the seven LR chromosomes (B1–B7), we show a dot plot comparison against the set of SR contigs that align, reporting their number in brackets

**Fig. 6:**
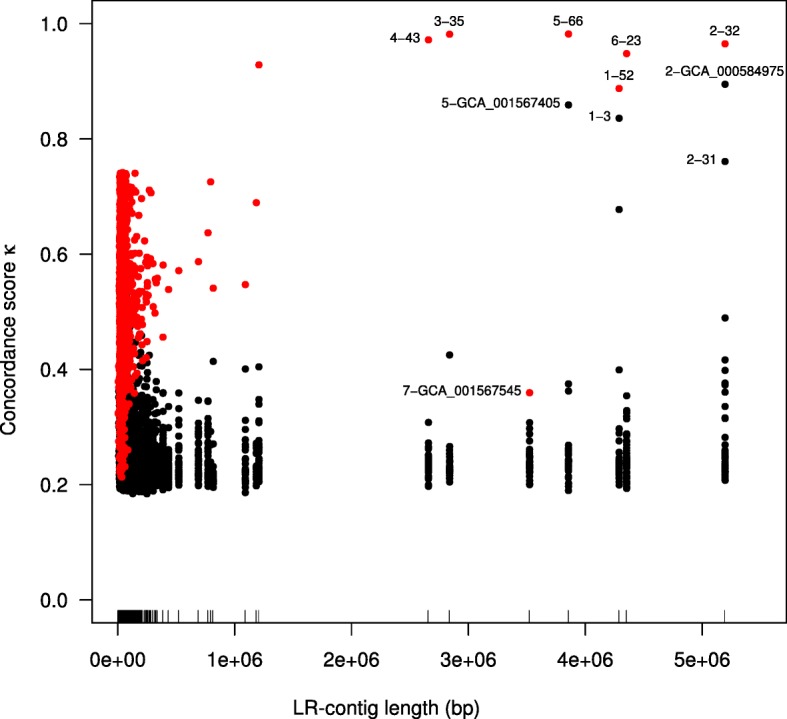
Overview of the concordance score for LR contigs, on the one hand, and SR-bins and reference genomes, on the other. The *x* axis shows the length of each LR contig, with the position of each tagged with a tick on the axis; the *y* axis shows the value of concordance score *κ*, and data points represent pairs of LR chromosomes and SR bins, or references genomes. Selected pairs with high concordance score are labeled with “ 〈LR-chromosome.id〉−〈SR-bin.id〉” for comparisons to SR-bins or “ 〈LR-chromosome.id〉−〈GCA_id〉” for comparisons to references. Within each set of the seven LR chromosome alignments, the pair with the maximum concordance score is shown in red. All LR chromosomes have highly concordant counterpart SR-bins, with the exception of LR chromosome 5. Further details on individual LR chromosomes are reported in Additional file 10: Figure S2

## Discussion

In this study, a single run of a nanopore MinION device on an enriched bioreactor community gave rise to a high coverage (384 ×) of the target polyphosphate accumulating organism, *Candidatus Accumulibacter*, but also 10–60 × coverage for 13 other taxa. From this data, in total, seven high quality draft genomes were obtained, six of which as closed circular chromosomes. Only three of these draft genomes have closely related reference genomes at NCBI. In all three cases, the LR chromosomes display a major improvement in continuity over the fragmented reference genomes, which were obtained by metagenomic assembly of short reads.

A potential concern might be that the reported megabase-sized contigs might be chimeric or otherwise incorrect. The results reported by CheckM and Prokka suggest that these sequences are entirely consistent with being complete bacterial chromosomes. Moreover, our comparison with a set of short reads sequenced from the same DNA provides further evidence that the reported LR chromosomes are correct, and that an extremely high degree of recapitulation is obtained when compared to draft genomes obtained from the same DNA extraction. However, it is possible that some parts of the reported LR chromosomes might locally represent a mixture of closely related strains.

One current issue with long-read sequencing technologies is that they produce a significant rate of erroneous insertions and deletions, which cause problems when performing translated alignments. Our work suggests that frame-shift aware alignment techniques can be used to reduce such problems. If short reads are available for the same DNA, then these can be used to polish the LR contigs so as to further reduce the frame-shift problem. On the data presented in this paper, short-read polishing reduced the average number of frame-shifts per kilobyte of aligned sequence to 1.2.

A major challenge for the use of long-read sequencing technologies in metagenomics is that the use of more aggressive DNA extraction techniques to access the DNA molecules of more robust cells may lead to more fracturing of the DNA molecules, which will limit the length of the sequencing reads. In this paper, our focus was on obtaining long enough reads to allow the assembly of complete chromosomes, so organisms present in low abundance or with more robust cells are underrepresented in the long-read data, as indicated in Additional file 9: Figure S1.

## Conclusions

This work suggests that it is now possible to obtain complete bacterial chromosomes from an enriched microbial community using Nanopore sequencing. We provide a straight-forward pipeline for processing such data. It performs assembly, alignment against NCBI-nr, taxonomic binning, frame-shift correction, bin quality analysis and annotation, in less than 6 h (see Fig. 2a).

The application of long-read sequencing techniques promises to allow the routine extraction of whole chromosomes from environmental samples, providing a much more detailed picture of individual members of a microbiome.

## Methods

### EPBR bioreactor

A sequencing batch reactor (SBR) with 5.4 L working volume was inoculated with activated sludge from an EBPR mother reactor. A slow feeding strategy was applied for the reactor operation, which has been shown to benefit the proliferation of *Ca.* Accumulibacter [21]. The SBR was operated in 6 h cycles, including 60 min feeding, 20 min anaerobic, 180 min aerobic, and a 100 min settling/decant stage. In each cycle, 2.35 L of synthetic waste-water composed of 0.53 L of solution A (containing 1.02 g/L NH_4_Cl, 1.2 g/L MgSO_4_7H_2_O, 0.01 g/L peptone, 0.01 g/L yeast extract, and 6.8 g/L sodium acetate) and 1.82 L of solution B (0. 312 g/L K_2_HPO_4_3H_2_O, 0.185 g/L KH_2_PO_4_, 0.75 mg/L FeCl_3_6H_2_O 0.015 mg/L CuSO_4_5H_2_O, 0.03 mg/L MnCl_2_, 0.06 mg/L ZnSO_4_, 0.075 mg/L CoCl_2_, 0.075 mg/L H_3_BO_3_, 0.09mg/L KI, and 0.06 mg/L Na_2_MoO_4_2H_2_O) (modified from [22]) was introduced into the reactor. The reactor was operated at 30 ^∘^C with an hydraulic retention time (HRT) and a solid retention time (SRT) of 12 h and 11 days, respectively. The pH was controlled at 7.00–7.60 with DO levels maintained at 0.8–1.2 mg/L during the aerobic phase. The SBR achieved P-release of 180–200 mg/L with complete P removal observed after a 6-month operation. The reactor was sampled on day 267 of the operation.

### DNA extraction

Genomic DNA was extracted from the sampled biomass with the FastDNA™SPIN kit (MP Biomedicals) for soil, using 2 × bead beating with a FastPrep homogenizer (MP Biomedicals). The DNA was then size-selected on a Blue Pippin DNA size selection device (SageScience) using a BLF-7510 cassette with high pass filtering with a 8 kb cut-off.

### Nanopore sequencing

The sequencing library was constructed from approximately 4 *μ*g of genomic DNA using the SQK-LSK 108 Ligation Sequencing Kit (Oxford Nanopore Technologies). Sequencing was performed on a MinION Mk1B instrument (Oxford Nanopore Technologies) using a SpotON FLO MIN106 flowcell (FAH85393) and R 9.4 chemistry, running for approximately 24 h. Data acquisition was performed using MinKNOW version 1.14.1 running on a HP ProDesk 600G2 computer (64-bit, 16 GB RAM, 2 Tb SSD HD) running Windows10. Base-calling was performed using Albacore version 2.3.1. Adaptor trimming was performed using Porechop [23] with default settings. This produced 694,955 reads of average length 9 kb (range 2 bp–66 kb). A summary of the long-read statistics is given in Additional file 1: Table S1.

### Long-read assembly

Long-read assembly was performed using Unicycler (v 0.4.6) running with default settings. Assembly of the 694,955 long reads produced 1702 LR contigs of average length 61 kb (1.3 kb-5.2 Mb). This took 104 wall-clock minutes (10.2 CPU hours) on a server. (All timings in this paper were measured on a server with AMD Opteron(TM) Processor 6274, 64 × 2.2 GHz, 512 GB memory). A summary of the long-read contig statistics is given in Additional file 2: Table S2.

### DIAMOND options for long reads

This paper introduces two new features in DIAMOND for use with error-prone long reads or contigs. First, the program now provides a *frame-shift mode* that performs frame-shift alignment of DNA sequences against a protein reference database [12]. This feature is activated using the command line option -F 15, which also sets the frame-shift dynamic programming penalty to a specific value, in this case 15.

Second, the program now provides the option to perform *range-culling*. This feature determines which alignments are reported to output. Without range-culling, the program reports the most significant alignments for the query, up to a given count or score, independent of their position along the query. With range-culling, the decision whether to report an alignment is made locally. By default, any alignment *A* found is reported, unless there exists another alignment *B* that covers at least 50% of *A* on the query and whose bit-score is significantly larger, by defaulting requiring that the score of *A* is less than 90% of the score of *B*. This feature is activated using the command line options --range-culling and --top 10.

### DIAMOND alignment

In preparation of running DIAMOND on the Unicycler LR contigs, we downloaded the NCBI-nr database in November 2018, obtaining 177.6 million protein reference sequences. DIAMOND required about 1 h to initially process the database.

DIAMOND was run on the set of LR contigs with the following options: --range-culling --top 10 -F 15 --outfmt 100 -c1 -b12 -t /dev/shm. The program required 140 walk-clock minutes (81 CPU hours) to align all 1702 LR contigs against the NBCI-nr database and obtain 1.8 million alignments for 1695 contigs.

In comparison, running DIAMOND on the LR contigs without using the long-read specific options take only 40 wall-clock minutes (15 CPU hours), but only finds 42,230 alignments, and is thus not useful in practice.

### Frame-shift correction

In Fig. 2b, we illustrate how to correct frame-shift errors in a given query DNA sequence, based on an alignment computed by DIAMOND in frame-shift mode. In a frame-shift alignment, a ‘/’ in the alignment transcript indicates that the aligner inreased the current frame of the query sequence by 1 at the given position, whereas a ‘∖’ indicates the current frame was increased by 1, as in http://last.cbrc.jp/doc/lastal.html. To perform frame-shift correction, in the former case, we insert a single unspecified nucleotide ‘N’ into the query sequence, whereas in the latter case, we insert two unspecified nucleotides ‘NN.’

To perform this correction on a long read or LR contig, we greedily select a maximal set of non-overlapping alignments for the whole query and use this set for correction. This is implemented in MEGAN.

### MEGAN analysis and frame-shift correction

The output file of DIAMOND was prepared for analysis with MEGAN using the program daa-meganizer, which is part of the MEGAN Community Edition suite, version 6.13.1. The following command line options were used:

--longReads --lcaAlgorithm longReads --lcaCoveragePercent 51 --readAssignmentMode alignedBases --acc2taxa prot_acc2tax-Nov2018X1.abin

The first three options select MEGAN’s long-read analysis mode and sets the amount of aligned sequence to be covered by a taxon during the LCA analysis to 51% [14]. The fourth option requests that the primary count associated with each taxon is the number of aligned reads contained in the contigs binned to that taxon. The final option instructs the program to use the November 2018 mapping of NCBI accessions to NCBI taxa. This “meganization” step took less than five wall-clock minutes (0.2 CPU hours).

A summary of the taxon bins obtained by MEGAN analysis is given in Additional file 3: Table S3.

Frame-shift correction was performed on all LR contigs using MEGAN’s Export Frame-Shift Corrected Reads … menu item, and the resulting sequences were saved into taxon-specific files, in just over 2 min.

### CheckM

The frame-shift-corrected bins were analyzed for their completeness and contamination using CheckM (v1.0.12) in lineage_wf mode. Data files for CheckM were downloaded on 26.11.2018 from https://data.ace.uq.edu.au/public/CheckM_databases. The full output of CheckM is provided in Additional file 4: Table S4.

### Prokka

We annotated the frame-shift-corrected bins using Prokka (v1.12) in metagenome annotation mode without specifying taxa. The taxonomic database for this version of Prokka is based on Rfam 1.12.

### 16S analysis

For all seven LR chromosomes, we extracted all 16S sequences annotated by Prokka and performed taxonomic classification of them using Silva [20], obtaining the correspondence between the MEGAN assignments and the Silva assignments (note that *Ignavibacteriaceae* appears within the *Chlorobi* group in the NCBI taxonomy) reported in Table 2.

All assignments were obtained using a threshold of 95% identity, except for the case of bin B4, where a lower threshold of 90% identity was needed to obtain an assignment.

### Comparison with genomic references

For each of the seven LR chromosomes, we determined the reference taxon that occurs the most times in DIAMOND alignments of the contig against NCBI-nr. We then aligned the LR chromosomes to the corresponding reference assemblies using Minimap2 [7] (v2.14-r883) with parameters -cx asm20 -t32 --secondary=yes -P. We found a significant level of DNA similarity in three cases, which we summarize here as dot plots (see Fig. 3). The other four LR chromosomes did not align to their corresponding reference sequences (less than 1% of the total chromosome covered by an alignment), or, indeed, to any genome in the whole of NCBI.

### Repeat analysis

We used Minimap2 to align all seven LR-chromosomes against themselves with parameters -cx asm10 -t32 --secondary=yes -P to find repeated regions in them. The option -c generates CIGAR strings in the output, -x asm10 is a preset of parameters for comparing assemblies with up to 10% divergence, -t32 sets the number of threads, --secondary=yes reports secondary alignments (by default Minimap2 reports only the best alignment), and -P retains all chains and attempts to elongate them. We then marked the positions that are within alignments of length equal to or greater than 500 in a contig to itself as repeat regions.

In order to check whether the repeat rates obtained for our contigs are typical for bacterial genomes, we performed the same analysis on all complete bacterial genomes in RefSeq (downloaded on 01.06.2018). Figure 4 suggests that the seven LR chromosomes have repeat-rates that are similar to those observed for complete bacterial genomes in RefSeq.

### Additional short-read sequencing

To support the evaluation of the long-read contigs, we performed additional short-read sequencing from the same sample. Genomic DNA library preparation was performed using a modified version of the Illumina TruSeq DNA Sample Preparation protocol. Sequencing was performed on an Illumina HiSeq 2500 using a read length of 301 bp (paired-end). The raw gDNA FASTQ files were processed using cutadapt (v 1.14) in paired-end mode (with default arguments except -overlap 10 -m 30 -q 20,20). We obtained 43,856,872 short reads in total. Summary statistics for the short reads are provided in Additional file 5: Table S5.

### Break-point and coverage analysis using short reads

We aligned all short reads against the LR contigs using Minimap2, with options -2 -f 0 -t 32 -F 10000 -ax sr --secondary=yes -N 10000. Then, considering each pair of reads, a valid clone, if the two aligned reads have the correct orientation with respect to each other and a distance below 800, we determined the clone coverage of each LR contig. Any stretch of LR contig, for which the clone-coverage is zero, is considered a potential break-point. We identified 11. All but one of these are covered by multiple long reads, and so we assume that they are not indicative of a long-read assembly error. The coordinates of the potential break-points are reported in Additional file 6: Table S6.

A comparison of the SR-coverage and LR-coverage of the 14 longest LR contigs reported in Table 1 yields a strong positive correlation (Pearson’s *R* = 0.9988), see Table 3.

**Table 3:** Comparison of LR and SR coverage

Bin	MEGAN assignment	Completeness	GC	LR	SR
		longest contig (%)	content (%)	coverage	coverage
B1	*Bacteroidetes bacterium* OLB12	95	43.6	57.3	117.5
B2	*Candidatus Accumulibacter* SK-02	94	61.3	384.2	707.8
B3	*Chlamydiia* (class)	94	38.2	48.8	107.6
B4	*Gammaproteobacteria* (class)	93	40.5	25.1	56.2
B5	*Bacteroidetes bacterium* OLB8	93	41.2	52.1	109.9
B6	*Rhodospirillales* (order)	92	63.6	29.5	56.1
B7	*Chlorobi bacterium* OLB5	88	38.1	38.7	90.2
B8	*Thauera* (genus)	14	68.9	32.7	60.5
B9	*Sphingobacteriales bacterium* 44-15	0	40.6	10.2	23.2
B10	*Bacteroidetes* (phylum)	32	43.5	14.1	27.7
B11	*Candidatus Contendobacter* B J11	19	62.6	15.4	22.3
B12	*Betaproteobacteria* (class)	10	62.9	37.1	66.0
B13	*Nitrospira* (genus)	27	60.4	17.6	28.7
B14	*Chloroflexi* (phylum)	8	51.8	13.3	18.9

For the longest contigs in each of the 14 bins reported in Table 1, we report the completeness as determined by CheckM, the average CG content, the average long-read coverage, and the average short-read coverage

### LR contig polishing using short reads

In the case that short reads are available, polishing of LR contigs using short reads will lead to a reduction of frame-shift error. To investigate this, we used pilon [24] (with minimap2 mapping of short reads to LR contigs as described above) to polish the LR contigs using our short reads. We then analyzed the polished LR contigs using DIAMOND + MEGAN and frame-shift correction, as described above. The resulting number of frame-shifts per kilobyte of aligned sequence was 1.2 (standard deviation 1.6), compared to 6 for unpolished LR contigs.

### Assembly of short reads

The 43.86 million short reads were assembled using SPAdes-3.12.0 [25] (default parameters except -meta -k 21,33,55,77,99,127 -t 30). We obtained a total of 539,404 short-read contigs (SR contigs) of at least 500 bp in length. See Additional file 5: Table S5.

### Comparison of *α* diversity between short and long reads

To compare the *α*-diversity represented in the short reads and SR contigs, on the one hand, and in the long reads and LR contigs, on the other, we used the program metaxa2 [26] to extract and taxonomically bin 16S sequences. We then computed the Shannon index based on the genus-level bins. The values for the short reads, SR contigs, LR reads, and LR contigs are 2.9, 3.9, 3.4, and 2.3, respectively. This indicates that the short-read dataset captures more diversity than the long-read dataset.

### Comparison of SR contigs and LR chromosomes

To verify the correctness of the seven LR chromosomes, we aligned them against the set of SR contigs using Minimap2, as described above in the section on repeat analysis, and present the results using dot-plots in Fig. 5. These plots indicate a perfect concordance between the LR chromosomes and corresponding SR contigs. (What appear to be breaks in four of the diagonals are artifacts due to “wraparound” in the circular chromosomes.)

For each of the seven LR chromosomes, we aligned all corresponding SR contigs against the corresponding reference genomes using Minimap2 (as described above) and find significant alignments only for SR contigs corresponding to the LR chromosomes 1, 2, and 5. This supports the conclusion that only three of the LR chromosomes are present in the current reference databases.

### Metagenomic binning of short-read assembly

Genome binning was performed on all SR contigs that were at least 2 kb in length using MetaBAT [27] (v2.12.1, using default parameters). This was followed by bin evaluation using CheckM (v1.0.11) (default parameters except lineage_wf -t 29). This gave rise to 80 bins, of which 21 (26%) fulfill the definition of “high quality” and 14 (18%) are considered “medium quality” [15]. We performed a CheckM analysis of these bins, and the result is reported in Additional file 7: Table S7.

We screened for 16S genes within the SR contigs using the USEARCH [28] module --search16s (v 10.0.240, 64 bit), and annotated these sequences using Silva.

In addition, for ease of comparison with the long read results summarized in Table 1, we also performed DIAMOND + MEGAN taxonomic binning of the SR contigs (using the same parameters as for the LR contigs, but without frame-shift correction), followed by CheckM analysis, and present the results in Additional file 8: Table S8.

### Measuring the concordance between SR bins and LR chromosomes

Here, we introduce the *concordance score*, which provides a measure of concordance between SR bins and LR contigs. In more detail, we used BLASTN [29] (version 2.4.0+, default parameters) to align all SR contigs (as queries) against all LR contigs (as subjects), retaining only the best hit for each pair of sequences. Based on this, for each SR bin and LR contig, we computed four scores:

- The average ratio of the alignment length to the length of the SR contig,
- The average sequence identity reported by BLASTN,
- The proportion of the LR contig that is covered by aligned SR contigs, and
- The proportion of the SR contigs in the bin that are aligned on the LR contig.

The concordance score *κ* is then defined as the mean of these four values. So, for a given LR contig, if we select an SR bin whose concordance score *κ* is close to 1, then that bin will consist mostly of contigs that tile the LR contig at a high level of sequence identity. We also use *κ* to measure the concordance between the contigs of a reference genome assembly and a LR chromosome.

### Comparison of SR bins and LR chromosomes

LR chromosome 1 is contained in the MEGAN taxonomic bin labeled *Bacteroidetes bacterium* OLB12. This LR chromosome is tiled by contigs from SR bin 52 (a medium quality “metagenome-assembled genome” (MAG), with a concordance score of *κ* = 0.88), and from SR bin 3 (*κ* = 0.84), which cover the first third and second two-thirds of the LR chromosome, respectively. SR bin 52 is annotated by CheckM to UID2570, which is selective for members of phyla *Chlorobi*, *Bacteroidetes*, and *Ignavibacteriae*, and thus taxonomically ambiguous. See Additional file 10: Figure S2a.

LR chromosome 2 is contained in the MEGAN taxonomic bin labeled *Candidatus Accumulibacter* sp. SK-02, and is tiled by contigs in SR-bin 32 (high quality MAG, *κ*=0.97). CheckM annotates this to lineage marker set UID3971, which is selective for *Accumulibacter*, *Dechloromonas* and *Azospira*, all contained in the Order of *Rhodocyclaceae*. See Additional file 10: Figure S2b. Examination of the alignments between LR chromosome 1 and the closely related SR-bin 31 (*κ*=0.76) shows that the contigs from SR-bin 31 fill a major gap in the coverage of LR-chromosome 1 by the members of SR-bin 32. See Additional file 10: Figure S2c. This suggests that SR-bin 32 and SR-bin 31 should be a single bin. The closest reference genome identified by MEGAN-LR is GCA_000584975.1 (*Candidatus Accumulibacter* sp. SK-02), with *κ* = 0.90.

LR chromosome 3 is contained in the MEGAN-LR taxonomic bin labeled *Chlamydiia* and is covered by contigs from SR-bin 35 (high quality MAG, *κ* = 0.98). SR bin 35 is annotated by CheckM to UID2982, which selects for members of phylum *Chlamydiae* and phylum *Verrucomicrobia*. We confirmed that LR chromosome 6 (and SR bin 35) are members of phylum *Chlamydiae* using a Minimap2 alignment against all extant reference or draft genomes in the PVC superphylum (data not shown). See Additional file 10: Figure S2d.

LR chromosome 4 is contained in the MEGAN-LR taxonomic bin labeled *Gammaproteobacteria* and is covered by contigs from SR-bin 43 (high quality MAG, *κ* = 0.97), which is annotated to *Gammaproteobacteria* by CheckM via UID4266. See Additional file 10: Figure S2e.

LR chromosome 5 is contained in the MEGAN-LR taxonomic bin labeled *Bacteroidetes bacterium* OLB8, is aligned to by SR-bin 66 (high quality MAG, *κ* = 0.98), which is annotated to *Bacteroidetes* by CheckM via UID2591. See Additional file 10: Figure S2f.

LR chromosome 6 is contained in the MEGAN-LR taxonomic bin labeled *Rhodospirillales*. This LR chromosome is tiled by contigs from SR-bin 23 (high-quality MAG, *κ* = 0.95), which is annotated to the order of *Rhodospirillales* by CheckM. SR-bin 23 contains a full length 16S sequence, which Silva assigns to the genus *Defluviicoccus* (a member of order *Rhodospirillales*). See Additional file 10: Figure S2g.

LR chromosome 7 is contained in the MEGAN-LR taxonomic bin labeled *Chlorobi bacterium* OLB5. While there is a good coverage of this LR chromosome by SR contigs, these are not contained in any SR-bin identified by MetaBAT. The closest reference genome, GCA_001567546, has a *κ* value of only 0.36. See Additional file 10: Figure S2h.

## Additional files

Additional file 1 **Table S1.** Summary of LR read data. (TXT 1 kb)

Additional file 2 **Table S2.** Summary of LR contig data. (TXT 1 kb)

Additional file 3 **Table S3.** Summary of LR contig taxonomic bins computed using DIAMOND + MEGAN-LR. (TXT 5 kb)

Additional file 4 **Table S4.** CheckM results for all 106 LR contig taxonomic bins. (TXT 24 kb)

Additional file 5 **Table S5.** Summary of short-read data. (TXT 1 kb)

Additional file 6 **Table S6.** Potential break-points in seven LR chromosomes, inferred as locations that have no short read clone coverage. (TXT 2 kb)

Additional file 7 **Table S7.** Summary of short-read assembly binning using MetaBAT. (TXT 7.94 KB)

Additional file 8 **Table S8.** SR assembly statistics and CheckM results for 14 taxonomic bins. (PDF 35.7 kb)

Additional file 9 **Figure S1.** Using Minimap2, we aligned all SR contigs against all LR reads and LR contigs. Here, we show, for a given level of average coverage of a SR contig by short reads, how many bases of the SR-contigs align to long reads only (“in LR”), or to LR contigs (“in LR contigs”), or not (“not in LR”). There are 221 SR contigs that have a coverage greater than 150 but are not shown in the plot. They cover 5.3 Mb in total, of which 49.6% is aligned to long reads and 50.4% to LR contigs. (PDF 20 kb)

Additional file 10 **Figure S2.** Concordance statistics for SR contigs against LR chromosomes. In each plot, the LR chromosome is represented by the *x* axis, and the five panels, from top to bottom, represent: (A) the locations of alignments to the LR chromosome, (B) the corresponding percent identity, (C) the alignment-length to query-length ratio, (D) the alignment length and (E) the query length. The colors red and black are used to distinguish between alignments to different SR-bins or reference genomes, as described in the text. (PDF 72 kb)

Additional file 11 **Figure S3.** Plot of LR contig length vs concordance score *κ*; highlighting pairs of LR chromosomes/contigs and SR bins or references that show high levels of concordance. (PDF 378 kb)
