# Supplementary material for: Annotated bacterial chromosomes from frame-shift-corrected long-read metagenomic data
Source: Microbiome. 2019 Apr 16;7:61. doi: 10.1186/s40168-019-0665-y (PMC6469205; doi:10.1186/s40168-019-0665-y)
Supplement: Supplementary file 8 — Table S8. SR assembly statistics and CheckM results for 14 taxonomic bins. (PDF 35.7 kb) [file 40168_2019_665_MOESM8_ESM.pdf]

	(a) DIAMOND+MEGAN taxonomic bin	(b) SPAdes SR-contigs	(c) Total (Mb)	(d) Average coverage	(e) CheckM Complete.	(f) Contam.
B1	*Bacteroidetes bacterium* OLB12	299	4.3	18	99	4
B2	*Candidatus Accumulibacter* SK-02	153	3.9	397	66	1
B3	*Chlamydiia* (class)	732	3.6	7	98	59
B4	*Gammaproteobacteria* (class)	3,399	7.2	4	82	77
B5	*Bacteroidetes bacterium* OLB8	106	3.7	93	98	1
B6	*Rhodospirillales* (order)	1,163	1.8	4	36	7
B7	*Chlorobi bacterium* OLB5	92	3.5	6	93	2
B8	*Thauera* (genus)	734	3.7	30	71	2
B9	*Sphingobacteriales bacterium* 44-15	770	3.3	15	82	4
B10	*Bacteroidetes* (phylum)	8,586	14.3	4	100	201
B11	*Candidatus Contendobacter* B J11	3,616	7.7	5	92	109
B12	*Betaproteobacteria* (class)	6,351	14.8	9	100	296
B13	*Nitrospira* (genus)	6,636	12.2	5	99	205
B14	*Chloroflexi* (phylum)	13,839	25.3	5	100	507
