# Supplementary figures and images for: Annotated bacterial chromosomes from frame-shift-corrected long-read metagenomic data
Source: Microbiome. 2019 Apr 16;7:61. doi: 10.1186/s40168-019-0665-y (PMC6469205; doi:10.1186/s40168-019-0665-y)

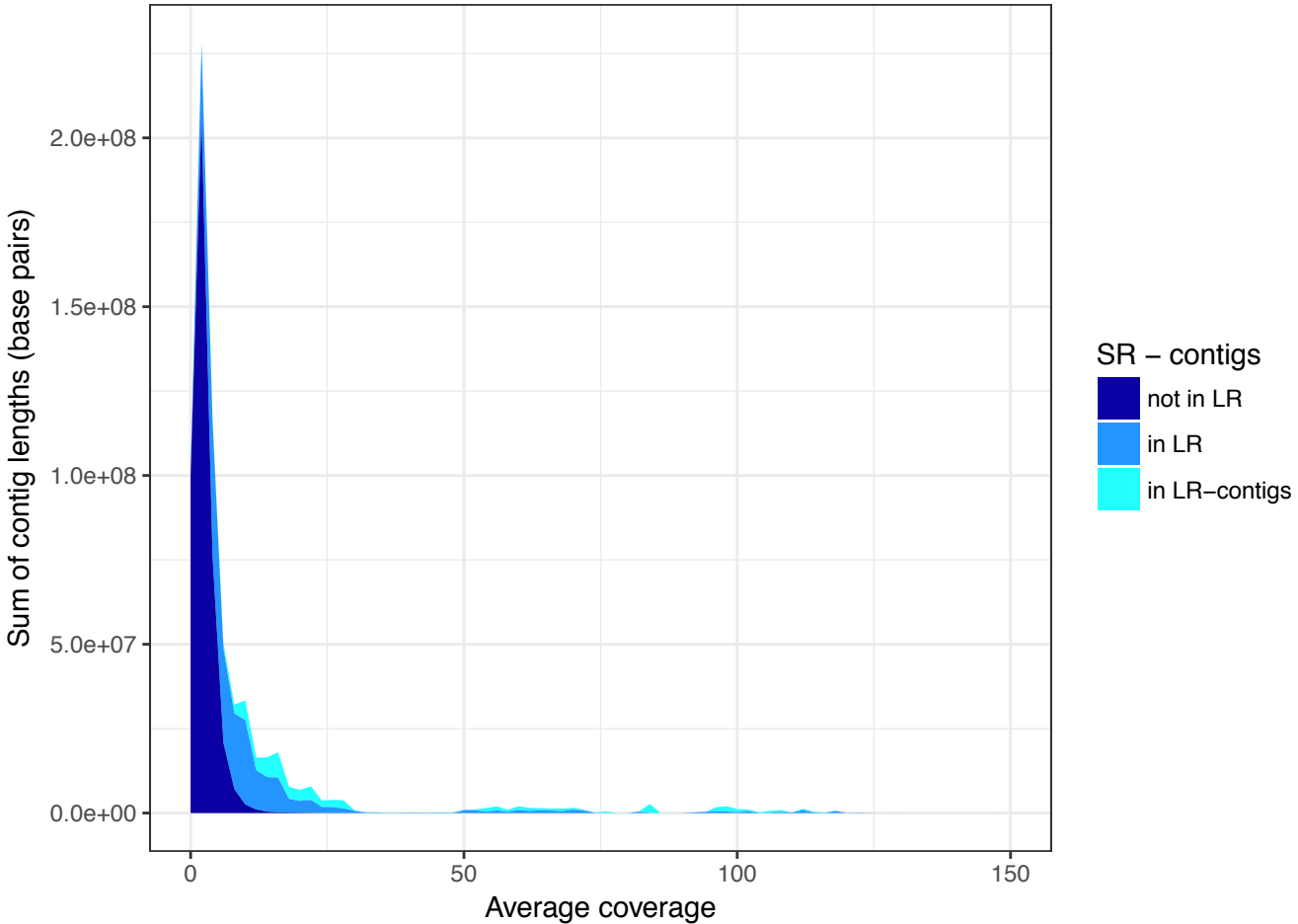

Supplement: Supplementary file 9 — Figure S1. Using Minimap2, we aligned all SR contigs against all LR reads and LR contigs. Here, we show, for a given level of average coverage of a SR contig by short reads, how many bases of the SR-contigs align to long reads only (“in LR”), or to LR contigs (“in LR contigs”), or not (“not in LR”). There are 221 SR contigs that have a coverage greater than 150 but are not shown in the plot. They cover 5.3 Mb in total, of which 49.6% is aligned to long reads and 50.4% to LR contigs. (PDF 20 kb) [file 40168_2019_665_MOESM9_ESM.pdf]

a

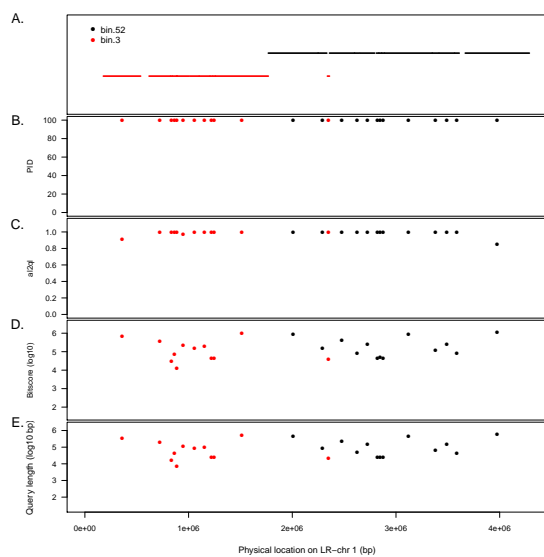

b

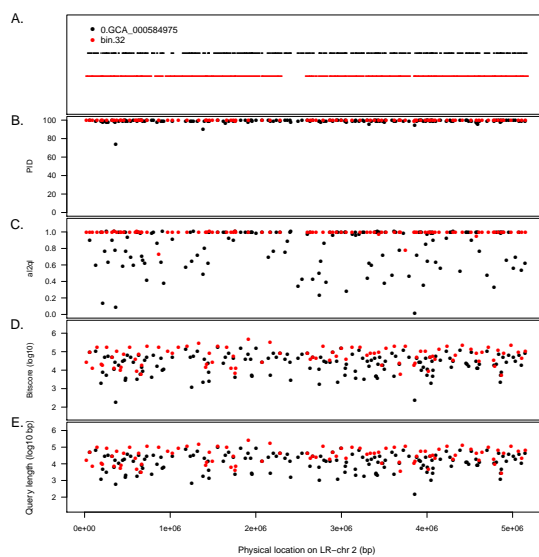

c

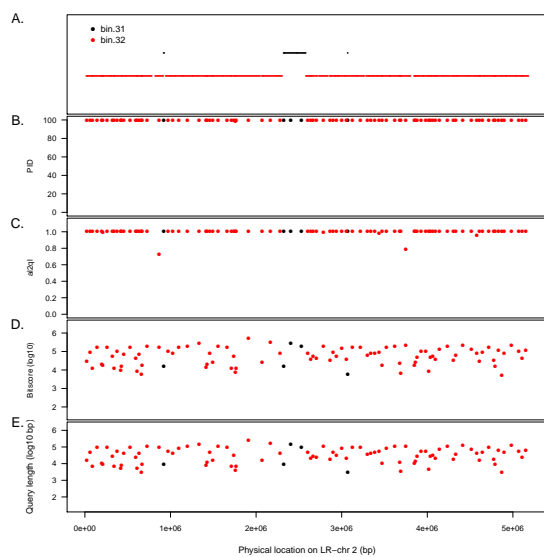

d

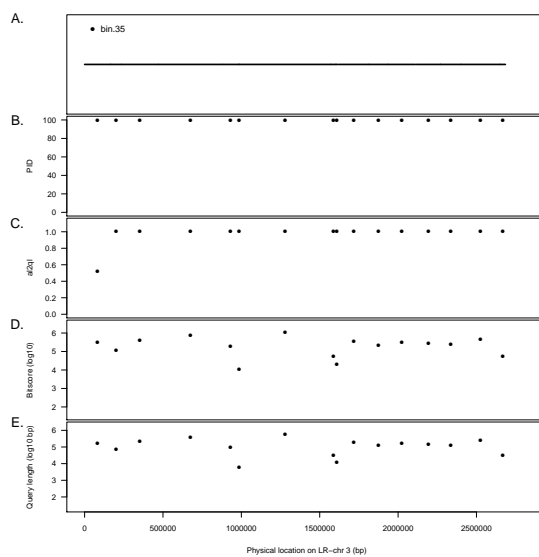

e

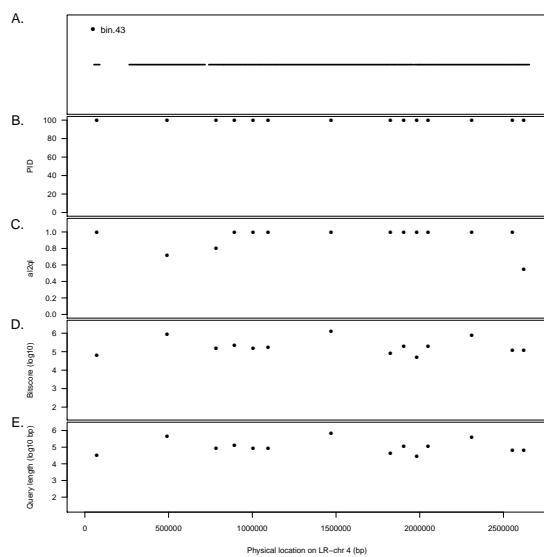

f

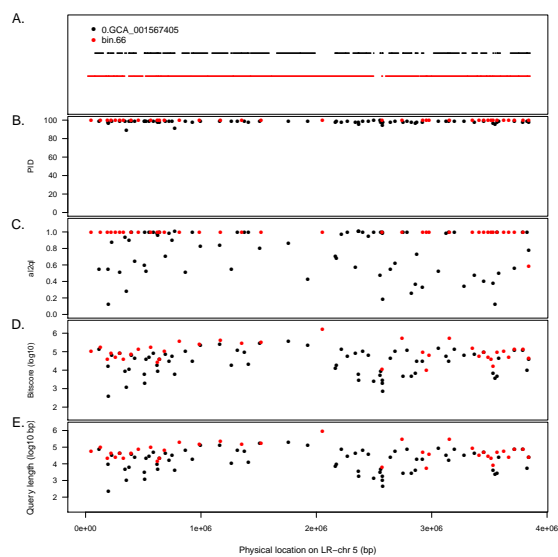

g

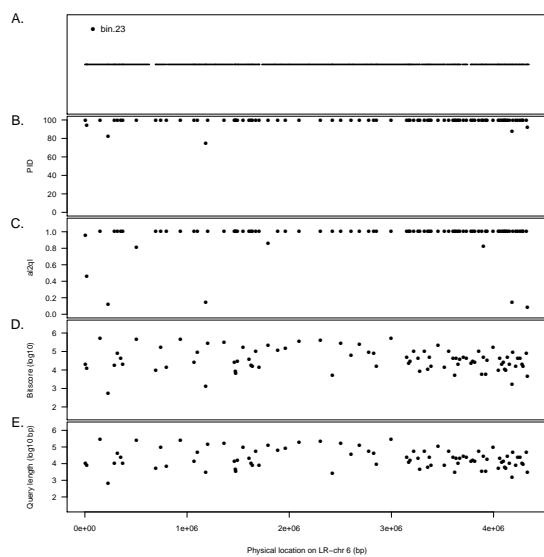

h

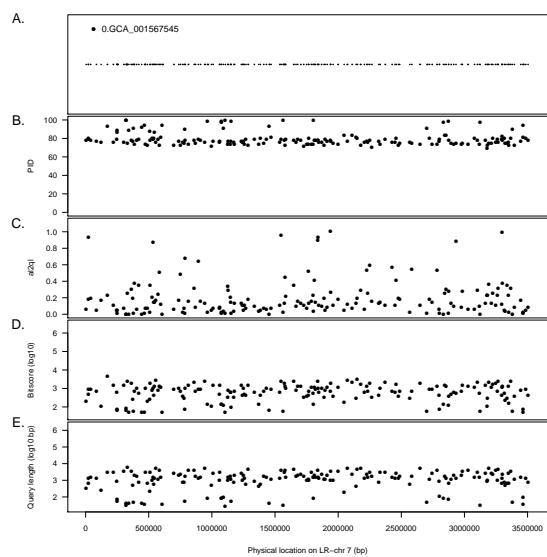

Supplement: Supplementary file 10 — Figure S2. Concordance statistics for SR contigs against LR chromosomes. In each plot, the LR chromosome is represented by the x axis, and the five panels, from top to bottom, represent: (A) the locations of alignments to the LR chromosome, (B) the corresponding percent identity, (C) the alignment-length to query-length ratio, (D) the alignment length and (E) the query length. The colors red and black are used to distinguish between alignments to different SR-bins or reference genomes, as described in the text. (PDF 72 kb) [file 40168_2019_665_MOESM10_ESM.pdf]

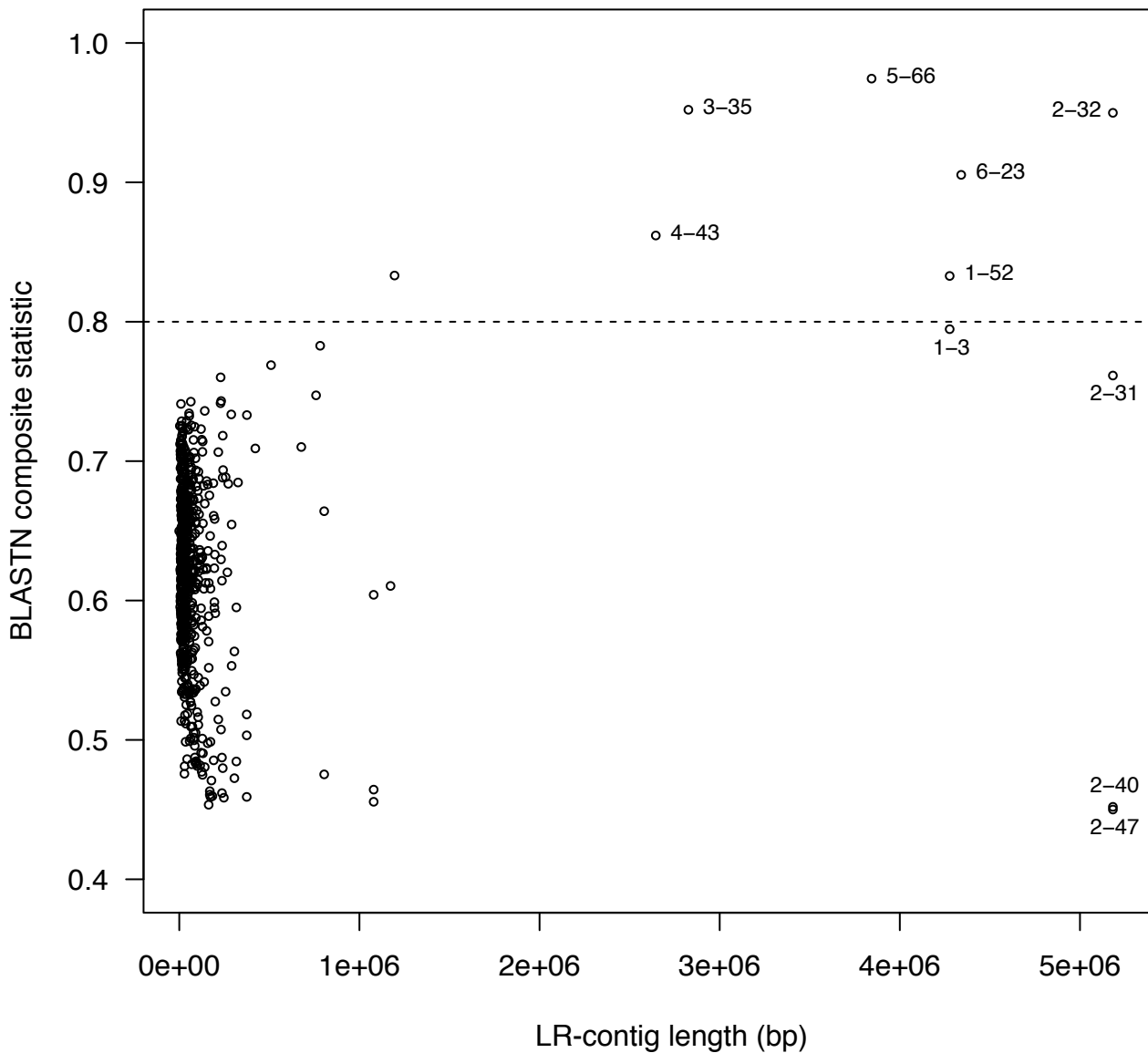

Supplement: Supplementary file 11 — Figure S3. Plot of LR contig length vs concordance score κ; highlighting pairs of LR chromosomes/contigs and SR bins or references that show high levels of concordance. (PDF 378 kb) [file 40168_2019_665_MOESM11_ESM.pdf]
